# TMPRSS2-ERG fusion protein regulates insulin-like growth factor-1 receptor (*IGF1R*) gene expression in prostate cancer: involvement of transcription factor Sp1

**DOI:** 10.18632/oncotarget.9837

**Published:** 2016-06-06

**Authors:** Shilhav Meisel Sharon, Yair Pozniak, Tamar Geiger, Haim Werner

**Affiliations:** ^1^ Department of Human Molecular Genetics and Biochemistry, Sackler School of Medicine, Tel Aviv University, Tel Aviv 69978, Israel; ^2^ Yoran Institute for Human Genome Research, Tel Aviv University, Tel Aviv 69978, Israel

**Keywords:** insulin-like growth factor-1 (IGF1), IGF1 receptor, TMPRSS2-ERG, prostate cancer, fusion proteins

## Abstract

Prostate cancer is a major health issue in the Western world. The most common gene rearrangement in prostate cancer is the TMPRSS2-ERG fusion, which results in aberrant expression of the transcription factor ERG. The insulin-like growth factor-1 receptor (IGF1R) plays a key role in cell growth and tumorigenesis, and is overexpressed in most malignancies, including prostate cancer. In this study we show that TMPRSS2-ERG mediates its tumorigenic effects through regulation of *IGF1R* gene expression. Silencing of T-ERG in VCaP cells resulted in downregulation of both IGF1R and Sp1, a critical *IGF1R* regulator. Co-immunoprecipitation assays revealed a physical interaction between transcription factors ERG and Sp1, with potential relevance in *IGF1R* gene regulation. In addition, transactivation of the *IGF1R* gene by ERG was mediated at the level of transcription, as indicated by results of promoter assays. To identify new co-activators of the TMPRSS2-ERG fusion protein we performed mass spectrometry-based proteomic analyses. Among other interactors, we identified AP-2 complex subunit mu (AP2M1) and caveolin-1 (CAV1) in association with ERG in cell nuclei. These proteins play a mechanistic role in IGF1R internalization. Our analyses are consistent with a potential novel function of TMPRSS2-ERG as a major regulator of *IGF1R* gene expression. Results may impinge upon ongoing efforts to target the IGF1R in the clinics.

## INTRODUCTION

The involvement of the insulin-like growth factor (IGF) axis in prostate cancer biology has been well established [1–3]. IGF1, both of endocrine as well as autocrine/paracrine origin, has been identified as a key player in the cellular and biochemical chain of events leading to transformation of the prostate gland. Accordingly, dysregulated IGF1 biosynthesis in transgenic mice with targeted expression of the ligand led to appearance of hyperplastic lesions resembling prostatic intraepithelial neoplasia [4]. The role of IGF1 in prostate cancer development is further supported by epidemiological studies showing an increase in serum IGF1 levels in patients who later developed the disease [5]. The physiological and pathological actions of IGF1 in the prostate gland are mediated by the IGF1 receptor (IGF1R), a tyrosine kinase-containing cell surface receptor with potent cell-survival and antiapoptotic activities [6, 7]. Most experimental and clinical evidence is consistent with the notion that acquisition of a malignant phenotype is an IGF1R-dependent process. However, its involvement in advanced stages of the disease is still controversial. It was previously shown that progression of prostate cancer from an organ-confined, androgen-sensitive disease to a metastatic, androgen-independent disorder is associated with a marked decrease in IGF1R expression [8]. Other studies, nonetheless, showed sustained up-regulation of IGF1R levels at advanced stage disease, including metastases [9, 10]. The mechanisms and pathways associated with regulation of *IGF1R* gene expression and action in prostate cancer remain largely unidentified.

Regulation of *IGF1R* gene expression is mainly attained at the transcriptional level [3, 11]. Comprehensive analyses of *IGF1R* promoter-binding nuclear proteins led to the identification of *cis*-elements and *trans*-acting factors that are responsible for the tightly regulated expression of the *IGF1R* gene in organ- and temporal-specific manners [12]. Transcription rate of the *IGF1R* gene is heavily dependent on a number of stimulatory nuclear proteins, including zinc-finger transcription factor Sp1 [13, 14], E2F1 [15], Krüppel-like factor-6 (KLF6) [16], High-mobility group AT-hook (HMGA1) [17], androgen receptor (AR) [18], etc. In addition, *IGF1R* biosynthesis is regulated by a number of negative transcriptional regulators, including p53/p63/p73 [19, 20], Breast cancer gene-1 (BRCA1) [21–23], Wilm's tumor-1 (WT1) [24–26], von-Hippel Lindau (VHL) [27], etc.

Tumor specific translocations that disrupt the architecture of transcription factors are a common theme in oncogenesis [28, 29]. These rearrangements create chimeras that are composed of modules derived from unrelated genes. Using a bioinformatic approach aimed at discovering candidate oncogenic chromosomal aberrations on the basis of outlier gene expression, Tomlins *et al* [30] reported the identification of recurrent gene fusions of the 5′ untranslated region of the *TMPRSS2* gene to the *ERG* or *ETV1* genes in prostate cancer. The *TMPRSS2* gene is located on chromosome 21 and is highly expressed in prostate epithelium. The gene encodes a 492-amino acid serine protease with five distinct domains, including a transmembrane region [31]. While the normal function of TMPRSS2 is unknown, the *TMPRSS2* gene has been identified as an androgen-responsive gene. Fusion of this gene to members of the ETS family of transcription factors, in particular *ERG* or *ETV1*, leads to overexpression of the oncogenes in a large portion of prostate cancer cases, but not in benign prostate samples, in an androgen-dependent manner. The downstream targets of ERG and ETV1 in prostate cancer have not yet been identified.

In view of the role of fusion protein TMPRSS2-ERG in prostate cancer and to expand our previous studies on the transcriptional regulation of the *IGF1R* gene, we examined the hypothesis that the *IGF1R* gene constitutes a novel downstream target for the TMPRSS2-ERG prostate-specific chimera. Results obtained revealed that (i) the fusion-encoded ERG oncogene is a potent *trans*-activator of the *IGF1R* gene; (ii) enhanced IGF1R expression is mediated at the level of *IGF1R* promoter transcription; and (iii) enhanced IGF1R expression leads to activation of cell-survival downstream signaling pathways. Accelerated *IGF1R* transcription was associated with elevated expression of zinc-finger transcription factor Sp1. In addition, using a mass spectroscopy proteomic approach we identified a series of ERG interactors that might be involved in ERG-mediated *IGF1R* transcription, processing and internalization.

## RESULTS

### Effect of T-ERG fusion protein expression on IGF1R levels

**Figure 1:**
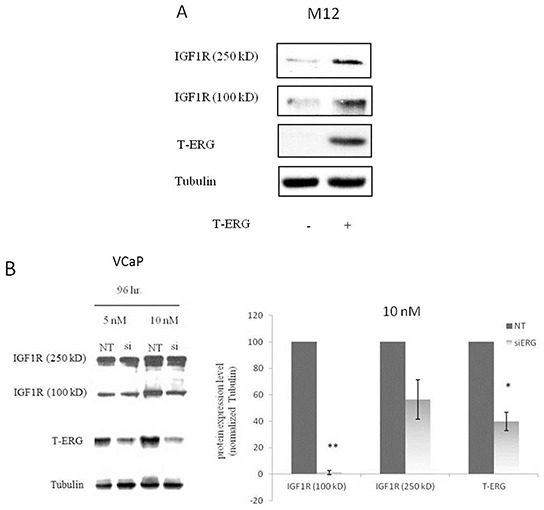

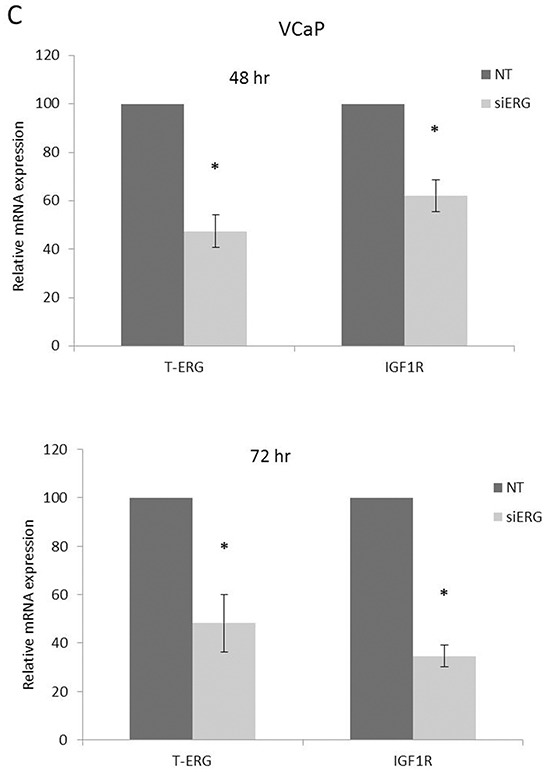
Effect of TMPRSS2-ERG on IGF1R protein and mRNA levels in prostate cancer cells **A.** M12 cells were infected with a T-ERG-encoding viral vector. Cells were lysed, electrophoresed through SDS-PAGE, followed by transfer and incubation with an IGF1Rβ subunit antibody. Both the mature (100-kDa) and precursor (250-kDa) forms of the IGF1R are displayed. **B.** VCaP cells were transfected with an siRNA directed against the fusion protein (siERG) at doses of 5 or 10 nM, or control non-targeting (NT) siRNA. Cells were harvested after 96 hr, and levels of T-ERG and IGF1R were measured by Western blots; *, p < 0.05 *versus* control; **, p < 0.01 *versus* control. **C.** VCaP cells were transfected with siERG (or NT) for 48 or 72 hr. Total RNA was then extracted and quantitative RT-PCR was performed using specific primers. Hsc70 primers were used to measure housekeeping gene hsc70 mRNA levels. *, p < 0.05 *versus* control (n=3 independent experiments).

The important role of IGF1R in prostate cancer initiation and progression has been well established. To investigate the potential effect of the TMPRSS2-ERG fusion protein on *IGF1R* gene expression, we employed two metastatic prostate cancer-derived cell lines with or without the chimera: the VCaP cell line, which expresses the chimeric protein in an endogenous manner, and the M12 cell line, which is devoid of the fusion protein. Infection of M12 cells with an ERG-encoding retroviral vector led to a marked increase in IGF1R levels in comparison to control (uninfected) cells (Figure 1A). Of interest, enhanced IGF1R levels were seen both in the precursor (~250-kDa) and mature (~100-kDa) IGF1R forms. To corroborate these results, ERG knockdown was performed in VCaP cells using an siRNA directed against the fusion protein (siERG) at doses of 5 and 10 nM, or non-targeting siRNA (NT) as control. As expected, the decreased T-ERG levels seen as a result of the siRNA treatments were associated with reduced IGF1R levels in comparison to controls (Figure 1B). Treatment with 10 nM siERG for 96 hr led to a 40-95% reduction in IGF1R levels. To examine the correlation between IGF1R protein and mRNA levels in response to T-ERG silencing, levels of IGF1R mRNA were measured by quantitative RT-PCR in siERG-transfected VCaP cells. ERG silencing led to 40% and 90% decreases in IGF1R mRNA levels at 48 and 72 hr, respectively, post-transfection (Figure 1C).

### Effect of T-ERG fusion protein expression on IGF1R downstream mediators

**Figure 2:**
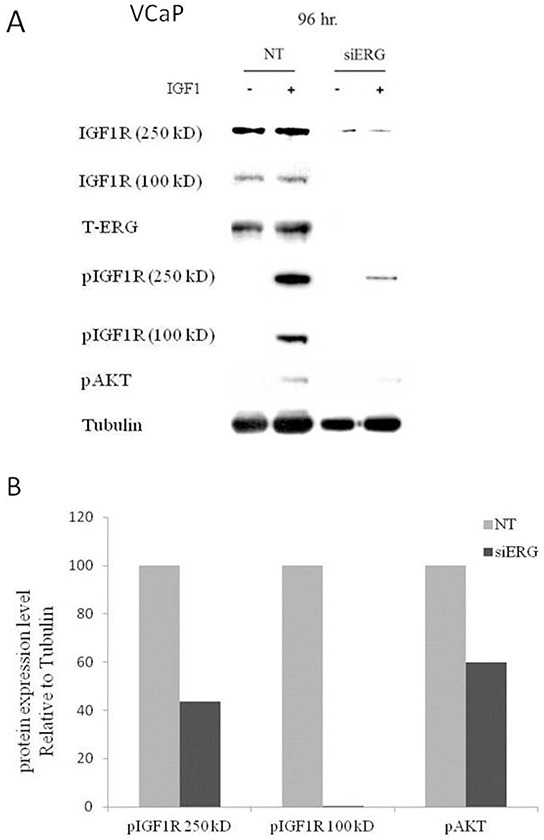
Effect of T-ERG expression on IGF1R downstream mediators **A.** VCaP cells were transfected with siERG (or NT, for control purposes) for 96 hr, followed by IGF1 treatment (50 ng/ml) during the last 10 min of the incubation period. Whole-cell lysates (100 μg) were resolved on SDS-PAGE and immunoblotted with antibodies against pIGF1R, T-IGF1R, T-ERG, pAkt, and tubulin. -, no IGF1 treatment; +, with IGF1 treatment. **B.** Scanning densitometry analysis of phospho-IGF1R and phospho-Akt. Bars represent phospho-protein levels of IGF1-treated cells normalized to tubulin values. Results of a representative experiment repeated three times with similar results are shown.

To assess the effect of the T-ERG fusion protein on IGF1-mediated signaling, siERG-transfected VCaP cells were treated with IGF1 for 10 min. Western blot analysis using an anti-phospho IGF1R revealed that ERG knockdown led to a 56% decrease in phosphorylation of the IGF1R precursor (250-kDa) and total abrogation of the phosphorylated mature IGF1R (100-kDa) (Figure 2). Moreover, a marked reduction (40%) was also observed in phospho-Akt levels upon siERG silencing. These results are consistent with a reduction in IGF1R activation and an overall decrease in IGF1-mediated downstream pathways activity.

### Effect of T-ERG fusion protein expression on *IGF1R* promoter activity

**Figure 3:**
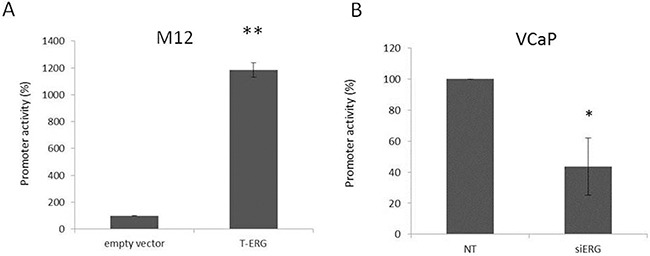
Effect of T-ERG on IGF1R promoter activity M12 **A.** and VCaP **B.** cells were cotransfected with 1 μg of p(−476/+640)LUC promoter reporter plasmid, along with 1 μg of T-ERG expression vector (or empty phCMV2) and 0.3 μg of pCMV β using the JetPEI transfection reagent. Luciferase and β-galactosidase activities were measured after 48 hr. Promoter activities are expressed as luciferase values normalized for β-galactosidase. Results are mean ± S.E.M. of 3 independent experiments, performed in duplicate dishes; *, p < 0.05 *versus* control; **, p < 0.01 *versus* control.

To examine whether T-ERG regulation of *IGF1R* expression is mediated at the level of transcription, M12 cells were transiently transfected with a proximal *IGF1R* promoter-luciferase reporter construct [p(−476/+640)LUC], containing 476 bp of 5′-flanking region and 640 bp of 5′-untranslated region, in the absence or presence of a T-ERG expression vector, along with a β-galactosidase vector. After 48 hr cells were harvested and luciferase and β-galactosidase activities were measured. As shown in Figure 3A, T-ERG expression led to a 23-fold increase in *IGF1R* promoter activity in comparison to cells transfected with an empty expression vector (phCMV2). To confirm the ability of T-ERG to control *IGF1R* gene transcription, promoter activity was assessed in siERG-transfected VCaP cells. As expected, T-ERG silencing led to a 60% reduction in *IGF1R* promoter activity (Figure 3B).

### Deletion analysis of T-ERG stimulation of *IGF1R* promoter activity

**Figure 4:**
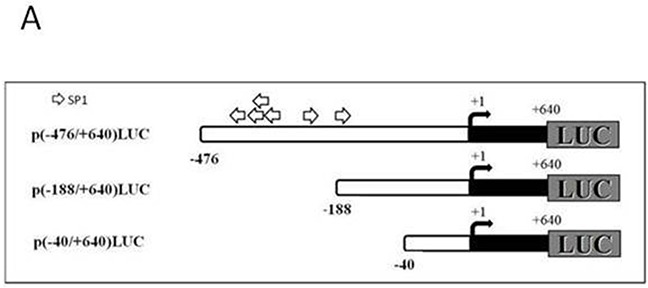

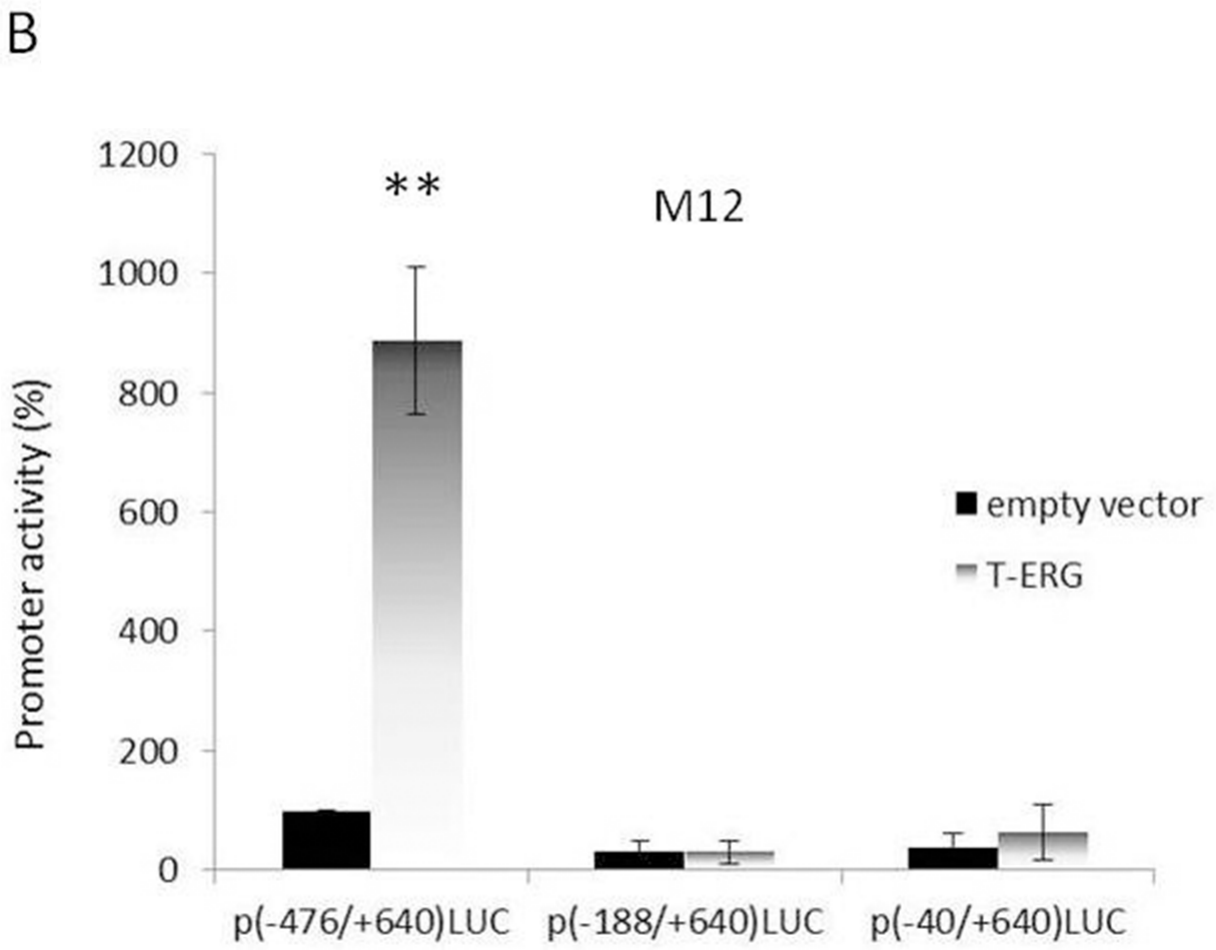
Deletion analysis of T-ERG effect on *IGF1R* promoter activity **A.** Schematic diagram of *IGF1R* promoter fragments used in transient transfection assays. Plasmids p(−476/+640)LUC, p(−188/+640)LUC, and p(−40/+640)LUC contain, respectively, 476, 188, and 40 bp of 5′-flanking region (open bar) and 640 bp of 5′-untranslated region (closed bar) of the *IGF1R* gene, fused to a luciferase cDNA (LUC). An arrow denotes the transcription ‘initiator’ element. The luciferase cDNA is not shown to scale. Open arrows denote a cluster of Sp1 sites. **B.** M12 cells were cotransfected with 1 μg of the indicated reporter plasmids, along with 1 μg of the T-ERG expression vector (or empty phCMV2) and 0.3 μg of pCMVβ using the JetPEI reagent. Luciferase and β-galactosidase activities were measured after 48 hr. Promoter activities are expressed as luciferase values normalized for β-galactosidase. Results are mean ± S.E.M. of 3 independent experiments, performed in duplicate dishes. **, p<0.01 *versus* control.

To identify the *IGF1R* promoter region responsible for mediating the effect of T-ERG, cotransfections were performed using promoter constructs with sequentially deleted 5′ flanking regions [p(−188/+640)LUC and p(−40/+640)LUC] (Figure 4A). Results of deletion analyses showed that the stimulatory effect of T-ERG was obliterated in cells transfected with shorter promoter fragments lacking the DNA region between nucleotides −476 and −188. Hence, our data indicate that sequences contained within this 5′-flanking fragment are critical for the stimulatory effect of T-ERG on *IGF1R* promoter activity (Figure 4B).

### Effect of T-ERG fusion protein on transcription factor Sp1

**Figure 5:**
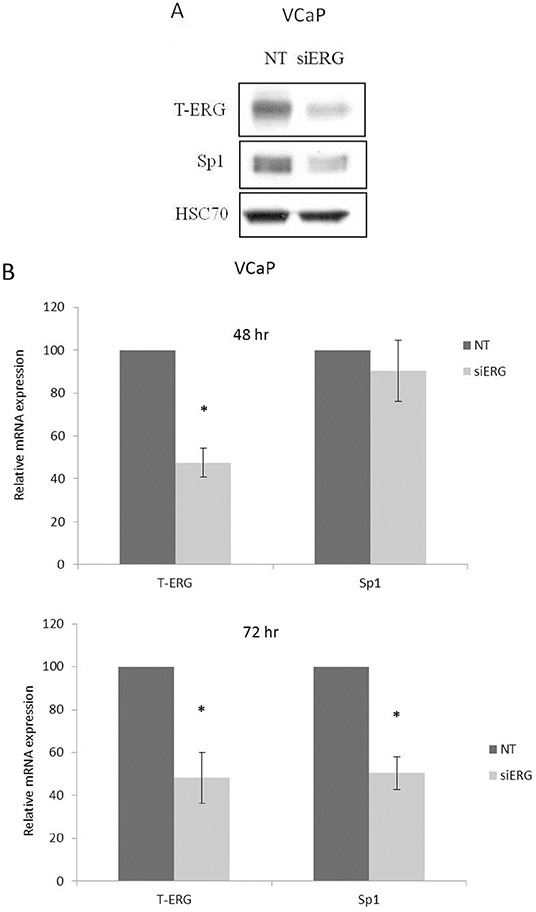

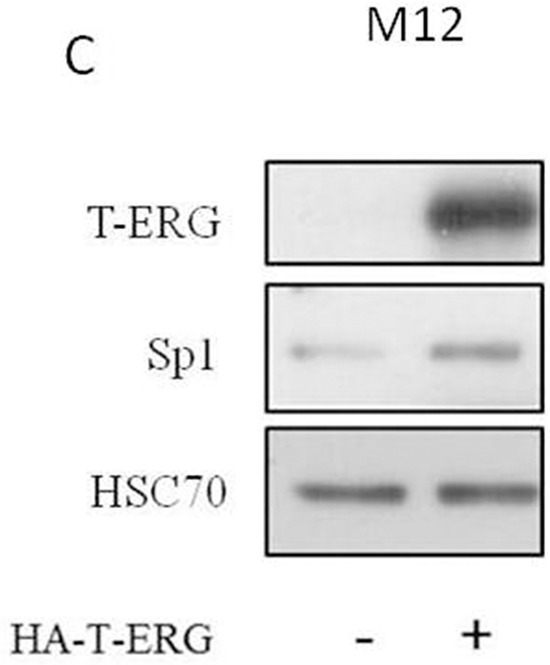
Effect of T-ERG expression on transcription factor Sp1 **A.** VCaP cells were transfected with siERG (or NT) for 96 hr. Whole-cell lysates (100 μg) were resolved on SDS-PAGE and immunoblotted with an anti-Sp1 antibody. Levels of hsc70 were used as a loading control. **B.** Total RNA was extracted from siERG-transfected VCaP cells at 48 and 72 hr and Sp1 mRNA levels were measured by quantitative RT-PCR. Hsc70 mRNA levels were measured for control purposes; *, p < 0.05 *versus* control (n=3). **C.** M12 cells were transfected with a T-ERG-expression vector (or empty phCMV2) and Sp1 levels were measured after 48 hr. Results of a representative experiment repeated three times with similar results are shown.

Previous studies have identified a cluster of Sp1 binding sites in the *IGF1R* proximal promoter region comprised between nucleotides −476 and −188 [13, 14]. Furthermore, zinc finger protein Sp1 expression was shown to be critical to achieve high levels of IGF1R mRNA and protein in most transformed cells. Hence, we examined whether TMPRSS2-ERG effect involves stimulation of transcription factor Sp1. To this end, siERG-transfected VCaP cells were harvested 96 hr post-transfection and Sp1 abundance was assessed by Western blot analysis. As shown in Figure 5A, T-ERG knockdown led to a 47% decrease in Sp1 levels. This reduction correlated with an 50% decrease in Sp1 mRNA levels at 72 hr post transfection (Figure 5B). Of interest, T-ERG knockdown did not affect Sp1 levels at 48 hr post transfection, implying that the effect of T-ERG on Sp1 level is most probably mediated *via* protein stabilization. In agreement, T-ERG-expression in M12 cells led to an increase in Sp1 levels (Figure 5C).

### Co-immunoprecipitation (Co-IP) assays of Sp1 and T-ERG

**Figure 6:**
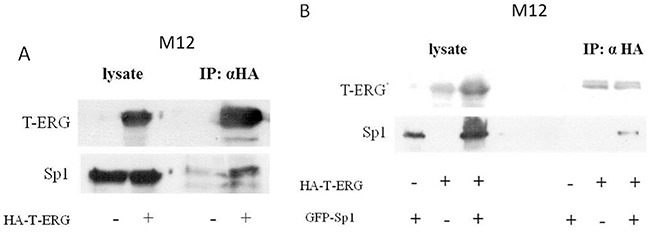
Co-immunoprecipitation assays of Sp1 and T-ERG M12 cells were transfected with 5 μg of a T-ERG expression vector (or empty phCMV2) **A.** or co-transfected with T-ERG expression vector and 5 μg of an Sp1 expression vector (or empty GFP vector) **B.** Cell lysates were co-immunoprecipitated using anti-HA (panels A and B). Results of a representative experiment repeated twice with similar results are shown.

To test for a possible physical interaction between Sp1 and T-ERG, co-IP assays were performed using extracts from M12 cells expressing an HA-tagged T-ERG (HA-T-ERG), with or without a co-expressed GFP-tagged Sp1 (pEGFP-Sp1). Results of co-IP assays showed that both the endogenous Sp1 protein (Figure 6A) as well as the expressed GFP-Sp1 (Figure 6B) was co-immunoprecipitated with HA-T-ERG by anti-HA antibodies. These data demonstrate that transcription factor Sp1 physically interacts with T-ERG. Unfortunately, attempts to detect an interaction between the endogenous proteins did not succeed.

### Mithramycin experiments

**Figure 7:**
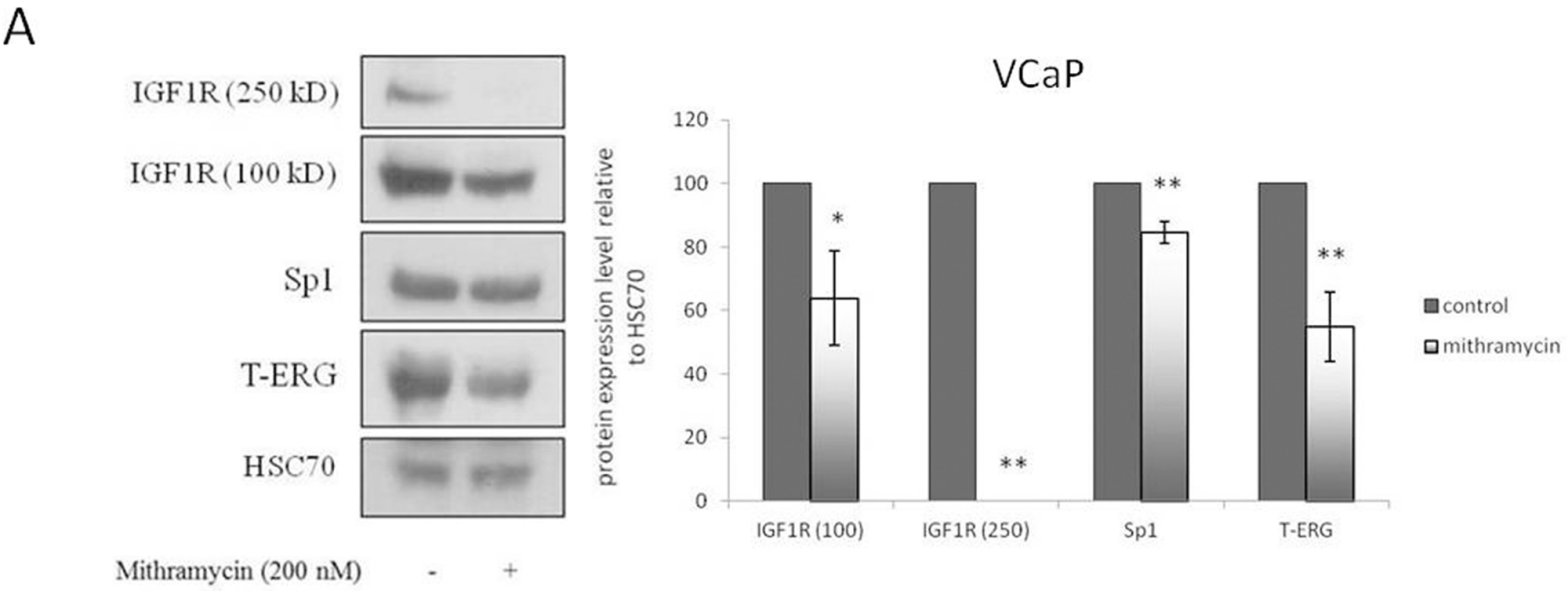

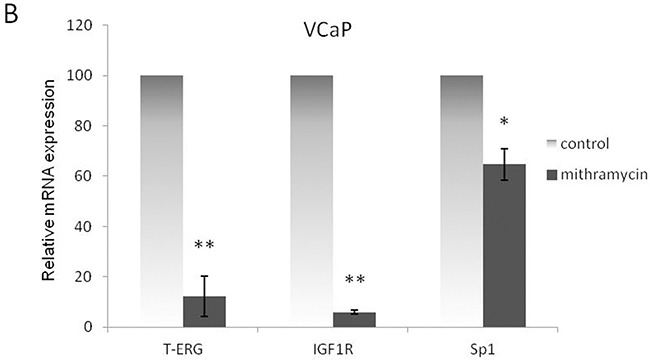
Mithramycin studies VCaP cells were treated with mithramycin for 24 hr. Protein levels were measured by Western blots **A.** and mRNA levels were measured by quantitative PCR. VCaP cells were treated with mithramycin for 24 hr. Protein levels were measured by Western blots **B.** Results are mean ± S.E.M. of 3 independent experiments, performed in duplicate dishes *, p < 0.05 *versus* control; **, p < 0.01 *versus* control.

The Sp1 inhibitor mithramycin was used to further analyze the role of Sp1 on T-ERG-dependent IGF1R overexpression. To this end, M12 cells were transiently transfected with a T-ERG expression vector, after which cells were incubated with 200 nM mithramycin for 24 hr. Results of Western blots indicate that the T-ERG-mediated upregulation of IGF1R levels was abolished by Sp1 inhibition. Protein levels, however, were largely reduced as a result of the mithramicyn treatment (data not shown). Nevertheless, a downregulation effect on T-ERG levels was observed in mithramycin-treated samples. To validate this results and to investigate whether Sp1 inhibition may affect endogenous T-ERG expression, VCaP cells were incubated with 200 nM mithramicyn for 24 hr and analyzed by Western blotting. Sp1 inhibition by mithramycin involves binding to GC-rich DNA sequences and displacement of transcription factor Sp1 from its binding sites. Accordingly, only a minor downregulation of Sp1 protein levels was observed (Figure 7A). Downregulation of IGF1R protein levels is consistent with Sp1 involvement in *IGF1R* gene regulation [13, 14].

Of interest, results obtained show that T-ERG protein levels were downregulated by 46% as a result of the mithramycin treatment. Data are consistent with either activation of TMPRSS2-ERG expression by Sp1 or, alternatively, stabilization of the chimera. To assess the effect of Sp1 activity on T-ERG mRNA expression levels, quantitative RT-PCR was performed on mithramycin-treated VCaP cells. Results showed that, in addition to its effect on IGF1R mRNA expression, mithramycin also reduced T-ERG mRNA levels (Figure 7B).

### Effect of TMPRSS2-ERG expression on IGF1R-directed targeted therapy

**Figure 8:**
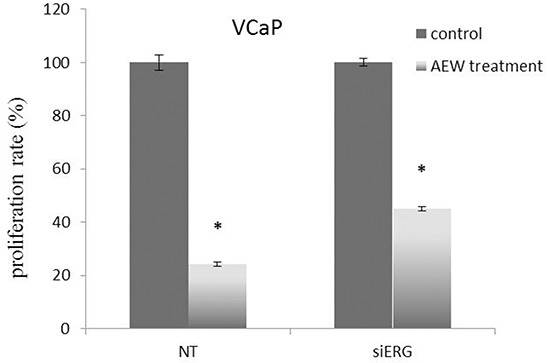
Effect of TMPRSS2-ERG expression on IGF1R-directed targeted therapy VCaP cells were transfected with 40 nM of siERG (or NT siRNA for control purposes). Forty-eight hours post-transfection cells were treated with the selective IGF1R inhibitor AEW541 for an additional 48 hr and proliferation rate was measured by XTT assays. Results are mean ± S.E.M. of 3 independent experiments. *, p < 0.05 *versus* control.

Given the key role of fusion protein TMPRSS2-ERG in regulation of *IGF1R* gene expression, we decided to evaluate next the hypothesis that IGF1R targeted therapy might be more effective in prostate cancer cells expressing the fusion protein in comparison to cells that do not express the chimera. To this end, siERG-transfected VCaP cells were treated with NVP-AEW541, a selective IGF1R inhibitor, followed by evaluation of proliferation rate. As shown in Figure 8, T-ERG-silenced cells were affected to a lower extent by the IGF1R inhibitor compared to T-ERG-expressing cells (55% vs. 75% inhibition). These results are consistent with an effect of TMPRSS2-ERG on the outcome of IGF1R-directed therapies.

### Proteomic analysis of TMPRSS2-ERG co-activators

**Figure 9:**
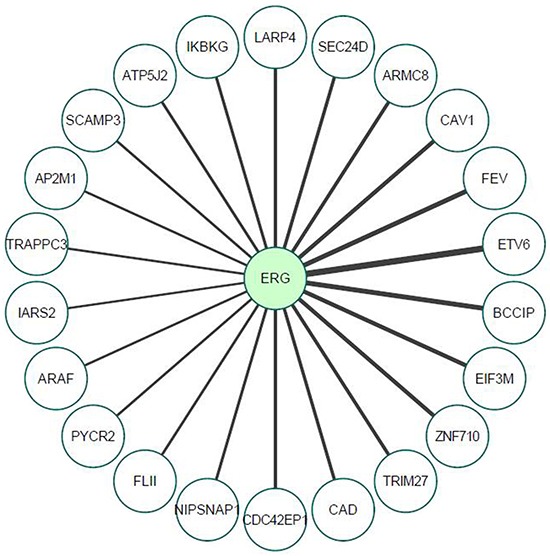
Proteomic analysis of TMPRSS2-ERG interactors, as determined by mass spectrometry M12cellswere transfected with a T-ERG expression vector (or empty phCMV2). Cells were harvested and total cell lysates were co-immunoprecipitated using an anti-HA antibody (co-IP assays were performed in triplicates) followed by mass spectrometry analysis. Line thickness represents Welch difference (see also Table 1).

In view of the fact that most transcriptional activators function as multiprotein complexes, and given our results showing that the effect of T-ERG on *IGF1R* gene expression involves activation of transcription factor Sp1, we decided to investigate the potential physical interactions of T-ERG in the specific context of *IGF1R* promoter regulation. In particular, we were interested in identifying T-ERG interactions that may be linked to transcription factor Sp1. To that end, we performed an immunoprecipitation assay in T-ERG-transfected M12 cells using an HA antibody (directed against the HA-tag encoded by the T-ERG expression vector), and anti-IgG as control. Mass spectroscopic analysis was performed to identify possible interactors. Overall, we identified 22 proteins that specifically interact with T-ERG compared to the control (Table 1 and Figure 9). Among these proteins we identified Fli1 and ETV6, which are known interactors involved in the transcriptional machinery [32]. Additionally, we found multiple proteins that are involved in protein transport and internalization (e.g., AP2M1, CAV1).

**Table 1:** List of TMPRSS2-ERG interacting proteins identified by mass spectroscopy-based proteomic analysis

Gene Name	Protein Name	Uniprot ID	Welch difference	Welch p-value
ETV6	Transcription factor ETV6	P41212	5.912296	0.019847
BCCIP	BRCA2 and CDKN1A-interacting protein	Q9P287	4.570442	0.001063
FEV	Protein FEV	Q99581	4.39208	0.026563
EIF3M	Eukaryotic translation initiation factor 3 subunit M	Q7L2H7	3.941612	0.000997
CAV1	Caveolin-1;Caveolin	Q03135	3.797178	0.004396
ZNF710	Zinc finger protein 710	Q8N1W2	3.739227	0.050713
ARMC8	Armadillo repeat-containing protein 8	Q8IUR7	3.284602	0.008734
TRIM27	Zinc finger protein RFP	P14373	3.202928	0.013209
SEC24D	Protein transport protein Sec24D	O94855	3.120237	0.02775
CAD	CAD protein;Glutamine-dependent carbamoyl-phosphate synthase;Aspartate carbamoyltransferase;Dihydroorotase	P27708	3.021694	0.002832
LARP4	La-related protein 4	Q71RC2	2.965799	0.005252
CDC42EP1	Cdc42 effector protein 1	Q00587	2.931694	0.025826
IKBKG	NF-kappa-B essential modulator	Q9Y6K9	2.732973	0.023957
NIPSNAP1	Protein NipSnap homolog 1	Q9BPW8	2.613799	0.042976
ATP5J2	ATP synthase subunit f, mitochondrial	P56134	2.555076	0.023341
FLII	Protein flightless-1 homolog	Q13045	2.406607	0.02791
SCAMP3	Secretory carrier-associated membrane protein 3	O14828	2.312815	0.008636
PYCR2	Pyrroline-5-carboxylate reductase 2	Q96C36	2.279021	0.010961
AP2M1	AP-2 complex subunit mu	Q96CW1	2.20255	0.009602
ARAF	Serine/threonine-protein kinase A-Raf	P10398	2.181118	0.003241
TRAPPC3	Trafficking protein particle complex subunit 3	O43617	2.154572	0.007609
IARS2	Isoleucine--tRNA ligase, mitochondrial	Q9NSE4	2.026957	0.003098

**Figure 10:**
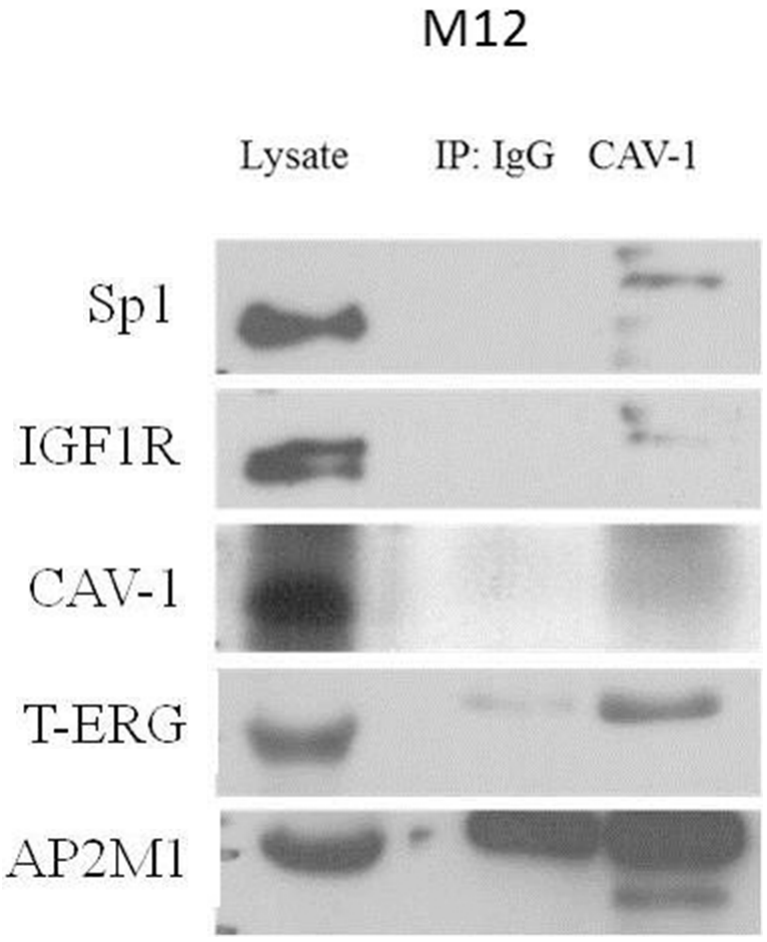
Co-immunoprecipitation assay of T-ERG with CAV1 and AP2M1 VCaP cells were harvested and lysates were co-immunoprecipitated using an anti-CAV1 antibody (or anti-IgG as control). Membranes were blotted with the indicated antibodies. Results of a representative experiment repeated twice with similar results are shown.

Of interest, different receptor tyrosine kinases, including IGF1R, are known to internalize through clathrin/caveolin-dependent pathways. Furthermore, clathrin or caveolin-1 (CAV1) inhibition blocked IGF1R internalization [33]. In line with this finding, two candidate interactors: AP-2 complex subunit mu (AP2M1) and CAV1 were selected for follow up analysis. To validate the physical interaction between CAV1, AP-2 and T-ERG co-immunoprecipitation assays were conducted using VCaP cells. As shown in Figure 10, T-ERG was co-immunoprecipitated with CAV1 by anti-CAV1, hence demonstrating that CAV1 physically interacts with T-ERG. In addition, AP-2, Sp1 and IGF1R were also co-immunoprecipitated with CAV1, implying the existence of a multimeric complex with yet unidentified role/s.

### Effect of TMPRSS2-ERG on cellular localization of IGF1R

**Figure 11:**
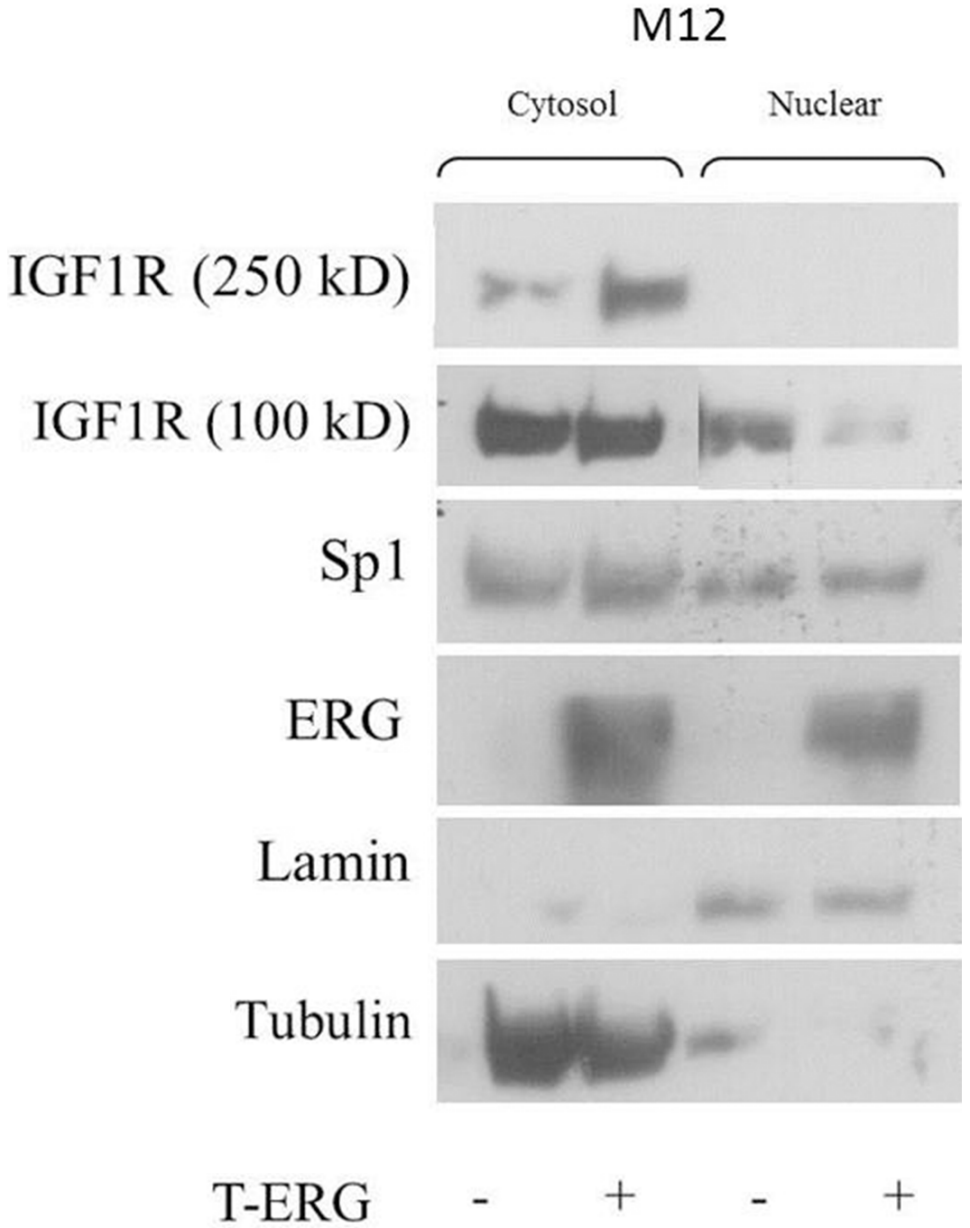
Effect of TMPRSS2-ERG on cellular localization of IGF1R M12 cells were transfected with a T-ERG expression vector (or empty phCMV2) for 48 hr. Then, cells were harvested and fractionated into cytoplasmic and nuclear fractions, followed by Western blot analysis. Results of a representative experiment repeated three times with similar results are shown.

Given the results of co-immunoprecipitation assays and in light of the fact that CAV1 and AP-2 participate in the IGF1R internalization process [33], we proceeded to examine the effect of T-ERG on IGF1R cellular localization. Fractionation assays were performed using T-ERG transfected M12 cells, followed by Western blot analyses. As shown in Figure 11, T-ERG overexpression led to a marked decrease in nuclear IGF1R levels.

## DISCUSSION

The identification of TMPRSS2-ERG as an important player in prostate cancer etiology had a major impact in basic and translational oncology. Recurrent chromosomal translocations leading to pathologic production of disrupted transcription factors are now recognized as a common event in adult epithelial tumors. The present study identifies the *IGF1R* gene as a novel downstream target for the TMPRSS2-ERG fusion protein in prostate cancer. Specifically, our analyses provide evidence of physical and functional links between frequent, cancer-specific, chromosomal rearrangements and the IGF1 signaling pathway, an important cell survival network. The chimeric protein TMPRSS2-ERG constitutes a prototype of a growing family of aberrant transcription factors harboring domains encoded by discrete genes [30]. For the most part, disrupted transcription factors exhibit gain-of-function activity that abrogates the intrinsic biological activity of each of the parental genes. In the specific case of TMPRSS2-ERG, the androgen sensitivity of the *TMPRSS2* promoter is responsible for the steroid-dependent expression of oncogene ERG in prostate epithelium, a key event in prostate carcinogenesis.

In the present study we showed that T-ERG expression in TMPRSS2-ERG-null M12 cells led to a major increase in IGF1R levels. Conversely, T-ERG silencing in TMPRSS2-ERG-containing VCaP cells led to a reduction in IGF1R expression. Decreased IGF1R levels were associated with a reduction in IGF1R activation as well as diminished phosphorylation of downstream target Akt. In terms of the mechanism of action of TMPRSS2-ERG, our data indicates that oncogene T-ERG stimulates *IGF1R* promoter activation. These results are consistent with a recent study which identified the IGF1R as a target of T-ERG action in prostate cancer cells [34]. On the basis of Mancarella's and our data, we aimed at elucidating the mechanistic aspects of the T-ERG-dependent IGF1R upregulation. Deletion analysis of the promoter allowed us to map a proximal promoter fragment, which seems to be responsible for the transcriptional effect of T-ERG. This promoter fragment, contained within nts −476 and −188 of the *IGF1R* 5′-flanking region, was shown previously to be extremely GC-rich (approximately 75% GC-content) and to contain a cluster of four Sp1 binding consensus sequences (GGGCGG). Sp1 was identified as a cardinal transactivator of the *IGF1R* gene in different tumor types, including prostate cancer [13]. Furthermore, Sp1 was demonstrated to play key roles in *trans*activation or *trans*repression of the *IGF1R* gene by a number of transcription factors, including the Wilm's tumor protein-1 [35] (WT1), estrogen receptor- [36], BRCA1 [21, 22], etc. We provide here evidence that T-ERG activates Sp1 expression, suggesting that the stimulatory effect of the fusion protein on *IGF1R* gene expression might be mediated, at least in part, via transcription factor Sp1. Of interest, the fact that inhibition of Sp1 action by mithramycin led also to a reduction in T-ERG levels is consistent with a bidirectional mode of action by which Sp1 levels are enhanced by TMPRSS2-ERG and, concomitantly, fusion protein expression and/or action are dependent on Sp1 expression.

Furthermore, proteomic analyses identified new ERG interactors including CAV1 and AP-2. Recent studies have demonstrated that IGF1R is internalized *via* clathrin- and CAV1-dependent mechanisms, and that both proteins are physically involved [33]. In support of our findings, a previous study has shown a physical interaction between IGF1R and CAV1 [37]. Data may imply an involvement of T-ERG in IGF1R internalization. We show, using fractionation assays, that the presence of T-ERG decreases nuclear IGF1R levels. Taken together, we envision a mechanism by which T-ERG enhances the IGF1R internalization process, leading to enhanced IGF1R activation. These novel ERG physical interactions may contribute to a stabilized cytoplasmic IGF1R and lower nuclear IGF1R levels.

The *IGF1R* gene has been identified as a downstream target of a series of disrupted transcription factors. EWS-WT1, the chimeric product of a recurrent t(11;22)(p13;q12) translocation that fuses the 5′ exons of the *EWS* gene to the 3′ exons of the *WT1* gene, constitutes the distinctive hallmark of desmoplastic small round cell tumor (DSRCT) [38–40]. Consistent with its oncogenic role, EWS-WT1 was shown to transactivate the *IGF1R* gene, unlike the WT1 component of the chimera which, in its wild type, untranslocated form strongly suppresses *IGF1R* transcription [26, 41]. Likewise, the PAX3-FKHR fusion protein characteristic of alveolar rhabdomyosarcoma was shown to transactivate the *IGF1R* promoter in sarcoma-derived cell lines [42]. The untranslocated PAX3 protein had a reduced potency. Taken together, regulation of *IGF1R* gene expression by aberrant transcription factors constitutes a common theme in oncology [43, 44]. The ability of unrelated disrupted gene products to control *IGF1R* transcription emphasizes the central role of this tyrosine kinase receptor as a critical regulator of cancer initiation and progression.

The regulation of the *IGF1R* gene by androgens has been recently explored using a series of isogenic prostate-derived cell lines and human xenografts [18]. We demonstrated that basal and phosphorylated IGF1R levels progressively decreased as prostate cancer cells became more tumorigenic and metastatic. In addition, we showed that wild type, but not mutant, AR along with dihydrotestosterone treatment increased *IGF1R* promoter activity and endogenous IGF1R levels. ChIP analysis showed enhanced AR binding to the *IGF1R* promoter in AR-overexpressing cells. Combined, we provided evidence that activated wild type AR enhances *IGF1R* transcription in prostate cancer cells *via* a mechanism that involves AR binding to the *IGF1R* promoter. AR mutations may alter the ability of the mutated protein to regulate *IGF1R* expression. The results of the present study provide evidence of an additional, indirect mechanism for androgen-mediated regulation of IGF1R levels. The physiological and pathological relevance of direct and indirect mechanisms for androgen regulation of *IGF1R* gene expression must be further explored.

In the pathophysiological context of prostate cancer, TMPRSS2-ERG fusion proteins are presumably functioning in the presence of the wild type, untranslocated ERG protein. The interactions between the translocation product and the full-length, untranslocated, ERG and, in particular, the impact of this interplay on *IGF1R* gene regulation must be further explored. As an anti-cancer target, the IGF1R axis has been studied in many clinical trials over the past years. However, most trials involving patients with adult tumors failed to show clinical benefit in the overall patient population. Our data show that the decreased proliferation rate achieved upon NVP-AEW541 IGF1R inhibitor treatment was less pronounced in ERG-silenced cells as compared to NT-transfected cells. If corroborated by larger clinical studies, these results may identify fusion protein TMPRSS2-ERG as a potential biomarker for IGF1R targeted therapy.

In conclusion, our results demonstrate that the *IGF1R* gene is a biologically relevant target for a novel family of prostate cancer-specific chimeric proteins. The net result of this chromosomal translocation is the *in-situ* production of oncogene ERG in an apparently androgen-dependent manner. Transactivation of the *IGF1R* gene by oncogene ERG constitutes a key event in prostate cancer development. Enhanced activation of the overexpressed IGF1R by locally produced or circulating IGF1 or IGF2 may provide a selective advantage to tumoral cells.

## MATERIALS AND METHODS

### Cell cultures

VCaP, a bone metastasis-derived cell line, was obtained from Dr. Raanan Berger (Sheba Medical Center, Israel). VCaP cells express the TMPRSS2-ERG chimera in an endogenous fashion. The VCaP cell line was maintained in DMEM medium containing 10% fetal bovine serum (FBS), 7.5% sodium bicarbonate, 11 mg/ml sodium pyruvate, 2 mM glutamine and 100 units/ml penicillin + 100 mg/ml streptomycin. Derivation of the M12 prostate cancer cell line has been previously described [45]. M12 cells are tumorigenic, highly metastatic and exhibit reduced IGF1 responsiveness. M12 cells do not express the TMPRSS2-ERG fusion protein. M12 cells were maintained in RPMI-1640 medium containing 10% FBS, 2 mM glutamine, 50 mg/ml gentamicin sulfate and 5.6 mg/ml fungizone. The M12 cell line was a gift of Dr. Joy L. Ware (Medical College of Virginia, Richmond, VA, USA). All reagents were purchased from Biological Industries Ltd., Kibbutz Beit Haemek, Israel.

### ERG overexpression studies

For stable transfections, human embryonic kidney 293T cells were cultured in DMEM medium with 10% FBS, 100 units/ml penicillin and 100 mg/ml streptomycin. The plasmids PWZL-tERG (Addgene, Cambridge, MA, USA) and PCL-amp (virus envelope) were transfected using the JetPEI® tranfection reagent (Polyplus Transfection Inc., Illkirch, France). Virus was harvested 48 hr post-transfection, filtered through a 0.45 μm pore membrane and Polybrene (4 μg/ml) (Sigma-Aldrich Co., St. Louis, MO, USA) was added to increase the efficiency of infection. To infect M12 cells with the retroviral vectors, cells were plated in 6-well plates two days before infection. Medium was removed and retrovirus was added. Virus-containing medium was removed 6 hr after infection and fresh cell culture medium was added. Three days post-infection cells from each two wells were removed into a 10-cm plate and selection was applied using 0.5 μg/ml blasticidin antibiotics. Stable cells were achieved approximately two weeks post-infection.

### ERG knockdown studies

For small interference RNA (siRNA) knockdown of ERG, siRNA against human ERG was purchased from Dharmacon Research Inc. (Lafayette, CO, USA). Negative control (non-targeting, NT) or siRNA against ERG was transfected into VCaP cells using INTERFERin® (Polyplus Transfection Inc.). Briefly, VCaP cells were seeded into 6-well plates the day before transfection, and 5 or 10 nM of siRNA and 6 μl of INTERFERin® were used for each transfection. siRNA knockdown of ERG was tested by immunoblot analysis.

### Plasmids and DNA transfections

For transient co-transfection experiments, genomic DNA fragments extending from nucleotides −476 to +640, −188 to +640, or −40 to +640 of the rat *IGF1R* gene (nucleotide 1 corresponds to the transcription start site) were subcloned upstream of a promoterless firefly luciferase reporter in the p0LUC vector [14, 24]. The T-ERG expression vector was kindly provided by Dr. Vera Magistroni (Università degli Studi di Milano Bicocca, Milan, Italy) [46]. The construct includes the coding region of the human *tERG* gene (T1/E4 variant), which was inserted into the phCMV2 vector. The T1/E4 variant, which is the tERG most common rearrangement, includes an untranslated region of the *TMPRSS2* gene fused to exon 4 of the *ERG* gene leading to an N-terminal truncated ERG protein that maintains its ERG DNA-binding domain. Since the first three *ERG* exons are lost during rearrangement, an alternative translation initiation site from an internal ATG codon is used to translate the fusion transcript. Briefly, the overexpressed T-ERG vector contains only coding regions of ERG and encodes a truncated ERG protein. M12 cells and VCaP cells were seeded in 6-well plates and transfected using the JetPEI®reagent, according to manufacturer's recommendations. Briefly, 1 μg of reporter plasmid, along with 1-1.3 μg of expression vector and 0.3 μg of a β-galactosidase expression plasmid (pCMVβ-, Clontech, Palo Alto, CA, USA) were used per plate. Two μl of transfection reagent were used per 1 μg of DNA. Promoter activities were expressed as luciferase values normalized for -galactosidase activity. For Sp1 expression experiments, the pEGFP-Sp1 vector was employed (Addgene).

### RNA isolation and quantitative RT-PCR

Total RNA was extracted with the PerfectPure RNA Tissue kit (5 PRIME, Gaithersburg, MD, USA) according to the manufacturer's protocol. High capacity cDNA kit (Applied Biosystems, Grand Island, NY, USA) was used to reverse transcribe RNA. Quantitative PCR was performed using an Applied Biosystems StepOne Plus Real Time PCR system with Fast SYBR® Green Mix.

### Western blot analyses

Cells were grown to confluence and then serum starved overnight. Ten minutes before harvest, cells were treated with 50 ng/ml of IGF1 (Cytolab Ltd., Rehovot, Israel). After incubation, cells were lysed in a protease-containing buffer. Protein content was determined using the Bradford reagent (Bio-Rad Ltd., Hercules, CA, USA) and bovine serum albumin (BSA) as a standard. Samples were electrophoresed through 10% SDS-PAGE, followed by blotting of the proteins onto nitrocellulose membranes. After blocking with 5% skim milk or 3% BSA, the blots were incubated overnight with the indicated antibodies, washed, and incubated with the appropriate horseradish peroxidase (HRP)-conjugated secondary antibody.

### Sp1 inhibition assays

In selected experiments, the Sp1-family inhibitor Mithramycin A (Sigma-Aldrich Co.) was added at a concentration of 200 nM 24 hr before cells harvest.

### Co-immunoprecipitation assays

Cell lysates were incubated overnight with 2 mg/ml of anti-HA or anti-CAV1 antibody at 4°C. The precipitates were then incubated with protein A/G agarose beads (Santa Cruz Biotechnology Inc., Santa Cruz, CA, USA) for 3 hr. Immunoprecipitates were pelleted by centrifugation at 2,500 rpm and then washed three times with a washing buffer. Finally, pellets were dissolved in 30 μl of sample buffer, boiled for 10 min, resolved on SDS-PAGE and immunoblotted with the indicated antibody.

### Fractionation of cytoplasmic and nuclear proteins

Cells were lysed with harvest buffer (10 mM HEPES pH 7.9, 50 mM NaCl, 0.5 M sucrose, 0.1 mM EDTA, 0.5% Triton X-100, 1 mM DTT, 10 mM tetrasodium pyrophosphate, 100 mM NaF, 17.5 mM beta-glycrophosphate, 1 mM PMSF, 4 μg/ml aprotinin and 2 μg/ml pepstatin A). The samples were centrifuged to pellet nuclei. The cytoplasmic fraction was transferred to a new tube. The nuclei was washed with buffer A (10 mM HEPES pH 7.9, 10 mM KCl, 0.1 mM EDTA, 0.1 mM EGTA, 1 mM DTT, 1 mM PMSF, 4 μg/ml aprotinin and 2 μg/ml pepstatin A) for 10 min. Nuclear proteins were lysed in buffer C (10 mM HEPES pH 7.9, 500 mM NaCl, 0.1 mM EDTA, 0.1 mM EGTA, 0.1% NP-40, 1 mM DTT, 1 mM PMSF, 4 μg/ml aprotinin and 2 μg/ml pepstatin A) for 15 min and transferred to a new tube, followed by the Western blot protocol.

### Proliferation assays

Cell proliferation was monitored using an XTT cell proliferation kit (Biological Industries Ltd.) according to manufacturer's instructions. Twenty-four hr post-siRNA transfection, VCaP cells were seeded at a density of 8,000 cells/ml in 96-well plates. Twenty-four hours after seeding, cells were treated with the NVP-AEW541 selective IGF1R inhibitor (2 μM) (Novartis Pharma, Basel, Switzerland) for 48 hr. Sample absorbance was measured with a spectrophotometer at a wavelength of 450-500 nanometers. Reference absorbance to measure non-specific readings was measured at a wavelength of 630-690 nanometers.

### Proteomic analyses of ERG interactors

Immunoprecipitation experiments were conducted as described above with the exception of the elution step. For MS analysis, after washing of the beads they were incubated for 1.5 h with 100 μl of elution buffer (2 M urea, 50 mM Tris-HCl pH 7.5, 1 mM DTT) and 1.5 μl sequencing-grade trypsin were added (stock 0.4 μg/μl, Promega). Beads were pelleted for two min at 1,500 rpm and the supernatant was transferred to a new tube. The process was repeated with a second elution buffer (2 M urea, 50 mM Tris-HCl pH 7.5, 5 mM iodoacetamide). After overnight incubation, 1μl of TFA was added and samples were desalted and concentrated on C18 Stage Tips [47]. Resulting peptides were separated by reverse-phase chromatography using a nanoflow HPLC system (EASY nLC1000; Thermo Fisher Scientific) coupled on-line to a Q-Exactive Plus mass spectrometer (Thermo Fisher Scientific) using a 140-minute linear gradient of water/acetonitrile. Raw MS files were analyzed by MaxQuant [48]. MS/MS spectra were searched against the human Uniprot database (published in May 2013) by the Andromeda search engine [49]. For identification, the false discovery rate (FDR) was set to 0.01 on the protein and peptide levels. Welch's t-test (FDR=0.05) between the T-ERG samples and control samples was used to determine possible interactors.

### Statistical analyses

The statistical significance of differences between groups in proliferation assays was assessed by Student's t-test (two samples, equal variance). p values < 0.05 were considered statistically significant.
